# Overweight, obesity and physical inactivity among women of reproductive age in Eastern Nepal: a cross-sectional community-based study

**DOI:** 10.1371/journal.pgph.0004360

**Published:** 2025-03-19

**Authors:** Prabina Bhattarai, Abhinav Vaidya, Thorkild Tylleskär

**Affiliations:** 1 Department of Global Public Health and Primary Care, Centre for International Health, University of Bergen, Norway; 2 Department of Community Medicine, Kathmandu Medical College, Kathmandu, Nepal; Universiti Malaya, MALAYSIA

## Abstract

Overweight, obesity, and physical inactivity have become more common in Nepal. The prevalence of overweight/obesity is higher among women than men, while the prevalence of physical activity is not well studied. This study aimed to understand the prevalence of overweight/obesity and physical inactivity among women of reproductive age in Eastern Nepal. We conducted a community-based cross-sectional study among women of reproductive age (18-49 years) in the Bhadrapur municipality, a sub-urban area of Eastern Nepal, in August-December 2019. We purposely selected six urban wards and approached 350 women using the probability proportional to size (PPS) sampling. In each ward, simple random sampling was used to identify participants. Height, weight, socio-demographic, and socioeconomic variables were collected, and body mass index (BMI) was calculated. Both a pedometer and a global physical activity questionnaire (GPAQ) were used to assess physical inactivity. Logistic regression in SPSS was conducted to identify the factors associated with overweight/obesity and physical inactivity. Out of 330 women, 41.9% were overweight/obese (BMI ≥ 25 kg/m^2^). Increased age (adjusted odds ratio (aOR) 1.052; 95% confidence interval (CI) 1.023-1.082) and being unemployed/housewives were positively associated with being overweight/obese. The prevalence of physical inactivity (≥ 5000 steps/day) was 13.9%. Manual workers had lower odds (aOR 0.282; 95% CI 0.080-0.989) of being physically inactive than unemployed women/housewives. The correlation between GPAQ and pedometer to measure physical activity was 0.35 (r² = 0.12). The high prevalence of overweight/obesity among women needs to be addressed. Women aged 40-49 years were at higher risk of being overweight/obese. Unemployed women/housewives were at a greater risk of being both overweight/obese and physically inactive. A huge variation in the physical activity levels was observed, which suggests a need for more studies on physical activity in a larger population with a broader age group and longer assessment periods.

## Introduction

Global data suggests an increasing trend in overweight and obesity, affecting 39% of the population in 2016 [1]. The concern of steadily increasing overweight and obesity corresponds to the steady increase in non-communicable diseases (NCDs) worldwide [2]. The prevalence of overweight and obesity is equally high in low-income regions, ranging from 21.9% in Southeast Asia to 31.1% in Africa [3]. This health situation is concerning since the cost associated with overweight and obesity is significant, with obesity alone accounting for 2.19% of the global gross domestic product (GDP) [4]. In low-income countries, the increasing prevalence of overweight and obesity contributes to a significant burden on the healthcare system.

In Nepal, the prevalence of overweight and obesity among adults has increased from 11% in 1996 to approximately 33.5% in 2022, with a change greater for women from 11.4% in 1996 to 35% in 2022 [5,6]. For women of reproductive age (15-49 years), the prevalence of overweight and obesity has surged dramatically, rising from 1.7% in 1996 to 22.2% in 2016 and is predicted to reach 64.5% in 2030 [7]. Wealthy, urban women and women belonging to advantaged ethnic/caste groups are more likely to be overweight and obese [7,8]. These factors are highly correlated with the food consumption patterns among women, which determines energy intake and potentially the risk of overweight and obesity [9,10].

Lack of physical activity is another factor associated with obesity and non-communicable diseases. The World Health Organization (WHO) defines lack of physical activity as less than 150 minutes of moderate-intensity activity per week or equivalent for adults [11]. Reports suggest that physical inactivity contributes to 6% of the disease burden from coronary heart disease, 7% from type 2 diabetes, 10% from breast cancer, and 10% from colon cancer, and contributes to 9% of premature mortality worldwide [12]. In Nepal, the prevalence of low physical activity assessed based on the WHO criteria had more than doubled among adults, with 3.5% in 2013 and 7.4% in 2019 [13,14]. In 2019, the prevalence of physical inactivity was even higher for women (8.2%) than for men (6.6%), which is much lower than the global average (21.1% for adult men and 31.7% for adult women) and even lower than for the Indian population (24.7% for adult men and 44% for adult women) [13]. Although these percentages are lower compared to other regions, the trend shows a higher proportion of physical inactivity in women, which is concerning as physical inactivity promotes overweight and obesity in women. Unfortunately, only a handful of studies have been conducted on physical inactivity in Nepal, most of which are centered around the capital city [15–17]. Additionally, none of the studies measured physical activity with any objective, reliable, and valid tool.

Considering the importance and the need to understand the burden and determinants of overweight/obesity and objectively measured physical inactivity level, we conducted a cross-sectional study in an urban area of Eastern Nepal. The objective was to determine the prevalence and risk factors associated with overweight/obesity and physical inactivity among women in Eastern Nepal.

### Subjects and methods

#### Ethics statement.

Ethical approval to conduct the current study was obtained from the Nepal Health and Research Council (NHRC) and the Regional Committee for Medical and Health Research Ethics (REC) in Western Norway (REK Vest. no 12204). All participants consented to collect the data and signed the consent form before participating in the study. Data collection was confidential.

#### Study design.

This was a community-based cross-sectional study conducted from August to December 2019 among women of reproductive age (18-49 years) in the Bhadrapur municipality, a sub-urban area of Eastern Nepal.

#### Study area.

Bhadrapur municipality is Nepal’s Eastern border with India, lying on a plain area with good access to transport. The estimated population size of the Bhadrapur was 71,000 in 2013, of which 49.6% were females. Out of the total population, 49,000 (69.4%) were above 18 years of age, and 22,000 (29.6%) were below 18 years old. Out of all women, 54% were of reproductive age (18-49 years), and 30% were children below 18 years. Bhadrapur municipality had six urban wards and four rural wards. The number of households was estimated to be 14,800 in an area of 97 km^2^, where 9,000 households were in the urban area and 5,800 households in the rural area.

#### Sample size.

We could not find the specific proportion of overweight and obesity among adult women in the urban plain region of eastern Nepal. Therefore, we used the national prevalence of overweight and obesity from the 2016 Nepal Demographic and Health Survey (NDHS) report for our sample size calculation [18]. We estimated the proportion of overweight and obese women to be 22% based on national data. While the NDHS provides representative data for adult women across Nepal, it is important to acknowledge potential disparities due to urban/rural and geographic differences. However, the estimate of 22% aligns with findings from a sub-analysis of the national data, which indicated that the prevalence of overweight and obesity in the urban areas of the eastern region ranges from 20-30% [19]. We calculated the sample size (95% confidence interval, a 5% margin of error, and a 10% non-response rate) as follows: sample size (N) = Z² ×p (1-p)/d² = 1.96² ×0.22 (1-0.22)/0.05² = 264. Allowing for non-response and assessment of risk factors using multivariable regression, we fixed the final sample size to 350.

#### Sampling.

**Fig 1:**
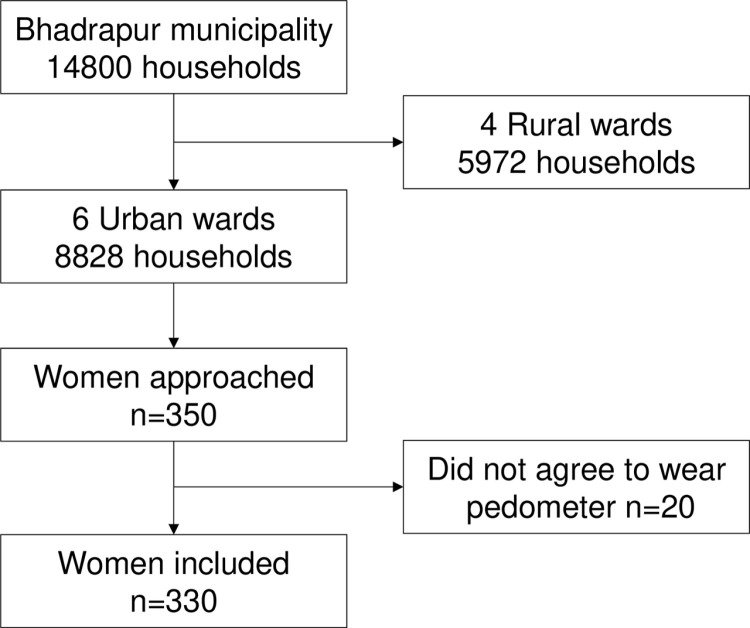
Flow diagram illustrating the process of participant selection in the study.

The steps for sample selection are shown in **Fig 1**. We purposely selected the urban area (the six urban wards) for our study. We wanted a representative sample of the urban area, and the number of participants from each ward was calculated using the probability proportional to size (PPS) sampling. To reach the requested sample size of 350, 48, 58, 69, 38, 63, and 74 households were selected from wards 5-10, respectively. In each ward, simple random sampling was used to identify participants. Based on the available list of households in each ward, sample households were selected using the Rand function in an Excel file. However, within ward number five, many people were unrecorded, and we doubted the correctness of the lists. Therefore, we switched to systematic sampling instead of simple random sampling in ward number five. With the help of the community leader in each village of ward number five, we determined the total number of houses. A random walk technique was used to find households. The first house by the main roadside, near the village border, was used as a starting point. The interval was selected by dividing the total households in each village by the required number of households. Household lists for two villages were made available, and there were eight other villages for which systematic sampling was necessary. Each selected household was visited to identify potential participants, and all the procedures were carried out in the participant’s home. One participant was selected per household, and if the participant was away for more than three days, she was replaced by a neighbor. In this study, a household was defined as a group of people who were living and eating their meals together for at least 6 of the 12 months preceding the study. Participants were recruited based on the following criteria:

- non-pregnant women in the reproductive age group (18-49 years)
- women living in the area for more than six months
- women who provided informed consent for the study

Written informed consent was obtained from the participants. Both the questionnaire and the medium of instruction were in the Nepali language. The data collection period extended from August 18, 2019 to December 13, 2019.

#### Inclusivity in global research.

Additional information regarding the ethical, cultural, and scientific considerations specific to inclusivity in global research is included in the supplementary checklist (S1 Checklist).

### Data collection

#### Anthropometric assessment.

Height was measured to the nearest 0.1 cm with a standardized wooden board following standard WHO protocols. Weight was measured to the nearest 0.1 kg using the Microlife WS50 digital weighing scale (Microlife AG Swiss Corporation, Windau, Switzerland) and calibrated every day using a standard weight. Participants were asked to wear a single-layer cloth during the measurement. Both measures were taken twice and reported in the questionnaire, and the average measurement was taken as the final value. A third measurement was done if the measurements differed by more than 0.5 kg or 0.5 cm. International BMI cut-offs were used to classify participants into underweight (BMI < 18.5), normal weight (18.5-24.99), overweight (25-29.99), and obese (≥ 30) categories. Asian BMI cut-offs were also used to classify participants into underweight (BMI<18.5), normal weight (18.5-22.99), overweight (23-27.49), and obese (≥ 27.5) categories. One of the major outcome variables of the study was overweight/ obesity.

### Physical activity assessment

Physical activity was assessed in two ways: subjectively using the WHO Global Physical Activity Questionnaire (GPAQ) and objectively using a pedometer. The GPAQ is the modified and improved form of the International Physical Activity Questionnaire (IPAQ), which has been used in physical activity research for decades [20]. In this study, the GPAQ was used to collect data on the type, frequency, duration, and intensity of physical activity during work, transportation, and leisure time in a fictional ‘typical week.’ Types of physical activity were categorized as occupational/domestic, transport-related, and leisure-time physical activity. We converted the data obtained from GPAQ to the metabolic equivalent of task (MET) minutes, defined as the energy cost at resting condition (1 kcal/kg/hour) [21]. The conversion of the GPAQ data to MET minutes was performed by multiplying the time spent in moderate-intensity activity by 4 and in vigorous-intensity activity by 8 [21].

For the objective physical activity assessment, we used a pedometer (Omron HJ320, China) to determine the average number of step counts per day. Step recordings went on until data was collected for three valid, consecutive days. A day was considered valid based on the self-reported wearing time of 10 or more hours. Women were categorized on a binary scale: ‘physically inactive’ (<5000 steps/day) or not, and on a graded scale: ‘physically inactive’ (<5000 steps/day), ‘low active’ (5000-7499 steps/day), ‘somewhat active’ (7500-9999 steps/day), ‘active’ (10000-11999 steps/day), ‘highly active’ (≥ 12000 steps/day) [20–22]. The primary outcome variable of the study was physical inactivity, which was defined by having step counts of less than 5000 per day.

### Questionnaire

The independent variables were assessed using a semi-structured questionnaire, including age, ethnic/caste group, religion, schooling years, occupational status, and ownership of household assets. A questionnaire was also used to generate a socioeconomic status (SES) based on ownership of assets, access to utilities/ infrastructure, and housing characteristics. The questionnaire was piloted in a small convenience sample for the feedback and comprehensibility of the questions.

Age in complete years and schooling years were recorded as continuous variables. Age in complete years was categorized into ‘18-29’, ‘30-39’, and ‘40-49.’ Schooling years were dichotomized into ‘Up to 9 years’ and ‘10 years and above.’ Marital status was categorized into two groups as ‘Married’ and ‘Other’ (Unmarried/ widow/ separated). Religion was categorized as ‘Hindu’ and ‘Other’ (Muslim/ Christian/ Buddhist/ *Kiranti*). Ethnic/ caste groups were categorized based on the caste classification card provided in the STEPwise approach to NCD risk factor surveillance (STEPS) survey 2013, Nepal [23]. Out of the six ethnic/caste groups provided in the caste classification card, two castes, ‘Upper castes’ and ‘Relatively advantaged *janajatis,’* were merged to form the ‘Advantaged ethnic/caste’ group. Similarly, the remaining four groups, which included disadvantaged *janajatis*, disadvantaged non-*Dalit* Terai caste groups, *Dalits*, and ethnic minorities/ Muslims, were merged to form a single different group called the ‘Disadvantaged ethnic/ caste group.’ Occupational status was recorded in three domains: ‘Non-manual’ (office workers/ self-employed/ students), ‘Manual’ (labor/ agriculture), and ‘Unemployed/housewives.’ Socioeconomic status was assessed based on household assets or access to utilities/infrastructure and housing characteristics, collectively including 31 items (included in the questionnaire). These 31 items were first checked for internal consistency, and only 25 items (Cronbach’s alpha = 0.914) were included for principal component analysis (PCA). The factor scores of the first principal component generated from the principal component analysis were utilized to create socioeconomic tertiles.

### Statistical analyses

Data were entered into Microsoft Excel and subsequently exported to IBM SPSS Statistics for Windows, version 25 (IBM Corp, Armonk, NY, USA) for analysis. Frequency analysis was used for all variables to check for consistency and possible data errors. Descriptive statistics, i.e., median, interquartile range, and percentages, were calculated for dependent variables. Prevalence estimates of BMI and step count categories were expressed as percentages. Histogram of BMI and step counts were created. Logistic regression with a backward conditional method was used to determine the factors associated with overweight/ obesity and physical inactivity using the adjusted odds ratios (aOR) and their 95% confidence interval (CI). For the interaction model between age group and physical activity level, the active group (10,000-11,999 steps/day) and the highly active group (≥ 12000 steps/day) were combined due to the low number of observations in the active group (n = 38). The bi-variable analysis was first conducted to identify possible factors associated with overweight/ obesity and physical inactivity. The multivariable analysis included variables with p < 0.10 as independent variables. All statistical tests were two-tailed, and the association was considered statistically significant when the confidence interval of the adjusted odds ratios did not cross the null value, i.e.,1. A Pearson correlation between step counts/day and MET-mins/day was calculated and presented as a scatter plot.

## Results

### Study participants

Out of the total 350 women, 20 did not agree to wear the pedometer and were excluded from the analysis (non-response rate = 5.7%) (Fig 1). Out of the 330 analyzed women, the majority were married (86.4%), more than half of the participants were unemployed/housewives (48.8%), 15.7% were engaged in a manual job, and 35.8% of women were in non-manual jobs (**Table 1**). The median age of the participants was 34 years with an interquartile range (IQR) of 14 years, and the median years spent in school was 10 years with an IQR of 7 years.

**Table 1:** Prevalence and risk factors of overweight and obesity in Eastern Nepal, N = 330.

Characteristics	N	Overweight or obese^1^ BMI ≥ 25 n (%)	Crude Odds Ratio (95% CI ^3^)	Adjusted Odds Ratio (95% CI ^3^)	Overweight or obese^2^ BMI ≥ 23 n (%)	Crude Odds Ratio (95% CI ^3^)	Adjusted Odds Ratio (95% CI ^3^)
Total	330	138 (41.8)			195 (59.1)		
*Model 1*							
Age (years) 18-29 30-39 40-49	118 109 103	27 (22.8) 57 (52.3) 54 (52.4)	1 3.76 (2.1-6.74) 2.56 (1.45-4.51)	1 4.20 (2.36-7.51) 3.51 (1.93-6.38)	48 (40.7) 75 (68.8) 72 (69.9)	1 3.21 (1.86-5.56) 3.39 (1.94-5.92)	1 2.86 (1.57-5.19) 3.35 (1.79-6.26)
Ethnic/ caste groups Disadvantaged^5^ Advantaged^6^	163 167	57 (35.0) 81 (48.5)	1 1.75 (1.13-2.73)		76 (46.6) 119 (71.3)	1 2.84 (1.80-4.47)	1 2.22 (1.37-3.62)
Marital status Other Married	45 285	11 (24.4) 127 (44.5)	1 2.5 (1.21-5.1)		17 (37.7) 178 (62.5)	1 2.74 (1.43-5.24)	1 2.1 (0.97-4.4)
Occupational status Manual (labor/agriculture) Unemployed/housewives Non-manual^7^	52 160 118	16 (30.8) 68 (42.5) 54 (45.7)	1 2.7 (1.3-5.6) 2.8 (1.3-5.9)	1 3.32 (1.59-6.96) 3.98 (1.82-8.69)	21 (40.4) 103 (64.4) 71 (60.2)	1 2.67 (1.4-5.1) 2.23 (1.15-4.34)	1 3.1 (1.53-6.2) 3.4 (1.59-7.3)
Schooling years Up to nine years Ten years and above	142 188	58 (40.8) 80 (42.6)	1 1.07 (0.69-1.7)		82 (57.7) 113 (60.1)	1 1.1 (0.71-1.72)	
*Model 2* ^&^							
Age (years)*PA levels 18-29*Sedentary 30-39*Low PA 30-39*Moderate PA 30-39*High PA 40-49*Low PA 40-49*Moderate PA 40-49*High PA			1 2.85 (1.27-6.43) 2.47 (1.12-5.44) 3.39 (1.54-7.46) 2.53 (1.04-6.12) 2.16 (0.96-4.86) 1.71 (0.83-3.52)			1 4.16 (1.6-10.82) 1.5 (0.69-3.29) 2.39 (1.06-5.39) 2.04 (0.81-5.1) 4.16(1.6-10.82) 1.63 (0.8-3.32)	
Age (years) 18-29 30-39 40-49	118 109 103	27 (22.8) 57 (52.3) 54 (52.4)	1 3.76 (2.1-6.74) 2.56 (1.45-4.51)	1 4.21 (2.36-7.51) 3.51(1.93-6.38)	48 (40.7) 75 (68.8) 72 (69.9)	1 3.21 (1.86-5.56) 3.39 (1.94-5.92)	1 2.86 (1.57-5.19) 3.35 (1.79-6.26)
Ethnic/ caste groups Disadvantaged^5^ Advantaged^6^	163 167	57 (35.0) 81 (48.5)	1 1.75 (1.13-2.73)		76 (46.6) 119 (71.3)	1 2.84 (1.80-4.47)	1 2.23 (1.37-3.62)
Marital status Other Married	45 285	11 (24.4) 127 (44.5)	1 2.5 (1.21-5.1)		17 (37.7) 178 (62.5)	1 2.74 (1.43-5.24)	1 2.05 (0.97-4.34)
Occupational status Manual (labor/agriculture) Unemployed/housewives Non-manual^7^	52 160 118	16 (30.8) 68 (42.5) 54 (45.7)	1 2.7 (1.3-5.6) 2.8 (1.3-5.9)	1 3.98 (1.83-8.69) 3.32 (1.59-6.96)	21 (40.4) 103 (64.4) 71 (60.2)	1 2.67 (1.4-5.1) 2.23 (1.15-4.34)	1 3.1 (1.53-6.19) 3.39 (1.59-6.2)

^1^Based on WHO international BMI cut-offs (BMI ≥ 25 kg/m^2^); ^2^Based on WHO Asian BMI cut-offs (BMI ≥ 23 kg/m^2^); ^3^CI = Confidence interval; ^5^All ethnic groups except upper castes and relatively advantaged *Janajatis;* ^6^Upper castes and relatively advantaged *Janajatis;* ^7^Non-manual includes self-employed, students and office workers. ^&^Only final model is shown. The initial model included physical inactivity, ethnic/caste groups and schooling years as well.

### Distribution of BMI and step counts/day

**Fig 2:**
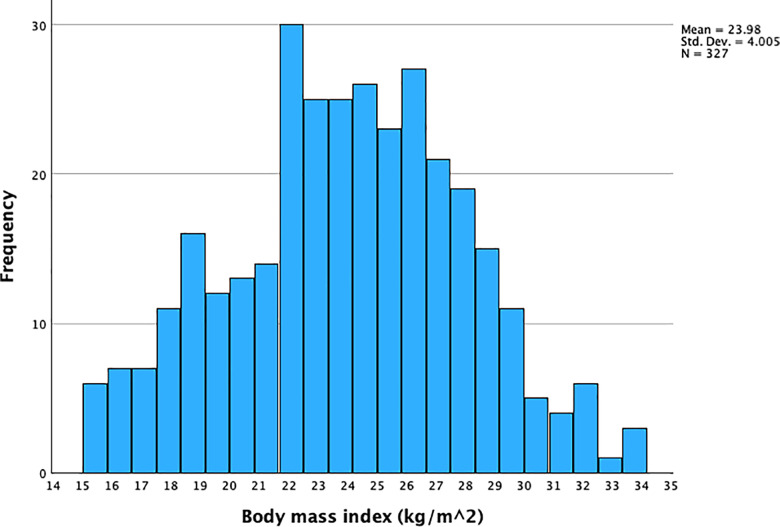
Distribution of body mass index in kg/m^2^ among 330 women of reproductive age in Nepal. Three upper-extreme observations in BMI were omitted in the figure.

**Fig 3:**
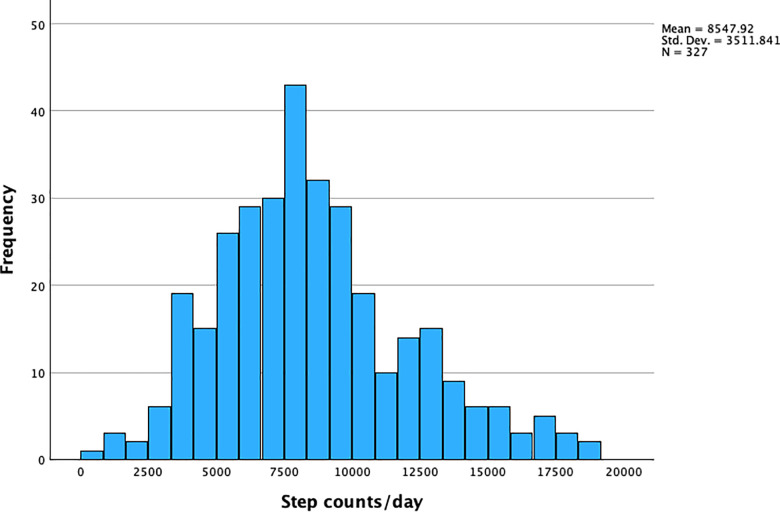
Distribution of step counts per day among 330 women of reproductive age in Nepal. Three upper-extreme observations in step counts/day were omitted in the figure.

The sample population (N = 330) had an approximately symmetric distribution of BMI with a median of 24.1 kg/m^2^ (**Fig 2**) and an interquartile range of 5 kg/m^2^. There was a widespread distribution of step counts/day with a median of 8150 step counts/day and an interquartile range of 4290 step counts/day (**Fig 3**).

### Correlation between MET-mins/day and step counts/day

**Fig 4:**
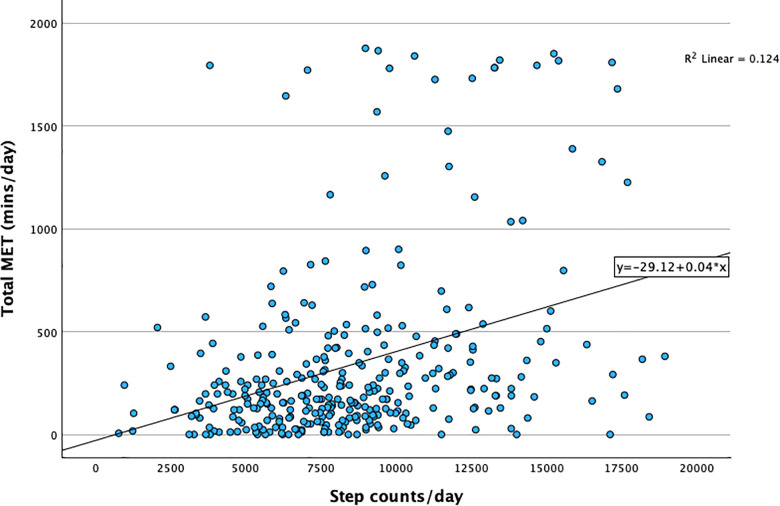
Correlation between step counts per day (by step count) and MET-minutes (by GPAQ questionnaire). Three upper-extreme observations in step counts/day were omitted in the figure.

The scatter plot in **Fig 4** depicts a weak correlation between the pedometer’s average number of step counts/day over 3 days and the average physical activity in MET-minutes/day (R = 0.35, p < 0.0001) in a fictional week. A slight increase in MET-minutes/day was observed with increasing step counts/day. However, the majority of data points were concentrated at the higher end of step counts/day, yet in the lower end of MET-mins/day range. The coefficient of determination (R^2^) was 0.12, which means that only 12% of the variation in the MET-minutes/day could be explained by the variation in step counts.

### Prevalence of overweight and obesity (WHO cut-off)

The overall prevalence of overweight or obesity based on the WHO BMI cut-off (BMI ≥ 25) was 41.9%. Out of them, a total of 35.2% were overweight (BMI ≥ 25 < 30), and 6.7% were obese (BMI ≥ 30). Women belonging to the age categories of 29-39 years (aOR 4.2; 95% CI 2.36-7.51) and 39-49 years (aOR 3.51; 95% CI 1.93-6.38) were approximately four times more likely to be overweight and obese compared to the women belonging to the age category of 18-29 years. Women in the unemployed/housewives’s (aOR 3.32; 95% CI 1.59-6.96) group and non-manual workers (aOR 3.98; 95% CI 1.82-8.69) had a significantly higher prevalence of overweight/obesity than manual workers (**Table 1 and** S1 Table).

### Prevalence of overweight and obesity (Asian BMI cut-off)

The overall prevalence of overweight and obesity based on Asian BMI cut-offs was 59.1%. Out of them, 38.8% were overweight (BMI ≥ 23 < 27.5), and 20.3% were obese (BMI ≥ 27.5). Women belonging to the age categories of 29-39 years (aOR 2.86; 95% CI 1.57-5.19) and 39-49 years (aOR 3.35; 95% CI 1.79-6.26) were approximately three times more likely to be overweight and obese compared to the women belonging to the age category of 18-29 years. Women in the unemployed/housewives’s (aOR 3.1; 95% CI 1.53-6.2) group and non-manual workers (aOR 3.4;95% CI 1.59-7.3) had a significantly higher prevalence of overweight/obesity than manual workers. Compared to disadvantaged ethnic/caste groups, women belonging to the advantaged ethnic/caste groups had significantly higher prevalence of overweight and obesity (aOR 2.22; 95% CI 1.37-3.62). Lastly, married women (aOR 2.1; 95% CI 0.97-4.4) were two times more likely to be overweight and obese compared to unmarried women (**Table 1 and** S2 Table).

### Interaction between age and physical activity on overweight and obesity (both WHO and Asian cut-off)

Model 2 indicated that there was a significant interaction between age and physical activity level on overweight and obesity based on the Asian BMI cut-off (p = 0.01) and WHO BMI cut-off (p = 0.008) in an unadjusted model (**Table 1**). With an increase in physical activity levels, women (40-49 years) showed a decrease in the prevalence of overweight and obesity compared to younger (18-29 years) sedentary women (**Table 1**). The interaction effect did not remain in the final model after applying the backward conditional method.

### Prevalence of physical inactivity

The prevalence of physical inactivity (defined as < 5000 step counts/day) was 13.9%. Of the participants, 25.8% had a low activity level (defined as 5000-7500 steps/day), while 15.8% were highly active (defined as ≥ 12,500 steps/day) (**Table 2**). The median step counts were high with a larger interquartile range (8150 ± 4290). Women in the unemployed/housewives’ group (aOR 4.73; 95% CI 1.07-20.94) and non-manual work group (aOR 3.54; 95% CI 0.77-16.4) had a significantly higher prevalence of physical inactivity compared to the manual work group. Women in the top socioeconomic tertile (aOR 1.77; 95% CI 0.84-3.77) showed a higher but non-significant level of physical inactivity than those in the lower socioeconomic tertile group (**Table 2 and** S3 Table). S4 Table shows the distribution of physical inactivity levels (based on MET minutes/week) by risk factors.

**Table 2:** Prevalence and risk factors of physical inactivity (< 5000 step counts/day), N = 330.

Characteristics	N	Physically inactive n (%)	Crude Odds Ratio (95% CI ^1^)	Adjusted Odds Ratio (95% CI ^1^)
Total	330	46 (13.9)		
Age (years) 18-29 30-39 40-49	118 109 103	18 (15.2) 17 (15.6) 11 (10.7)	1 1.03(0.5-2.11) 0.66 (0.29-1.48)	
Ethnic/ caste groups Disadvantaged^2^ Advantaged^3^	163 167	19 (11.6) 27 (16.2)	1 1.46 (0.78-2.75)	
Marital status Others Married	45 285	10 (22.2) 36 (12.6)	1 0.51 (0.23-1.11)	
Occupational status Manual (labor/agriculture) Unemployed/housewives Non-manual^4^	52 160 118	2 (3.8) 28 (17.5) 16 (13.6)	1 5.30 (1.22-21.1) 3.92 (0.87-17.7)	1 4.73 (1.07-20.94) 3.54 (0.77-16.4)
Schooling years Up to nine years Ten years and above	142 188	15 (10.6) 31 (16.5)	1 1.67 (0.86-3.23)	
Socioeconomic tertiles Lowest Middle Top	110 110 110	13 (11.8) 9 (8.1) 24 (21.8)	1 0.66 (0.27-1.63) 2.08 (0.99-4.34)	1 0.60 (0.24-1.49) 1.77 (0.84-3.77)

^1^CI = Confidence interval; ^2^ All ethnic groups except upper castes and relatively advantaged *Janajatis.*

^3^Upper castes and relatively advantaged *Janajatis;* ^4^ Non-manual includes self-employed; students and office workers.

## Discussion

This study in the plain region of sub-urban Eastern Nepal showed a high prevalence of overweight and obesity among reproductive-aged women - 41.8% based on WHO cut-off and 59.1% based on Asian BMI cut-off. Being overweight and obese was strongly associated with age, ethnic/caste group, and occupational status. This was the first study in Nepal to use an objective physical activity tool to measure physical inactivity in suburban women, and in this group, more than 14% of women were physically inactive (< 5000 steps/day). Physical inactivity was associated with occupational status.

The prevalence of overweight women and obesity in our study was considerably higher than the national prevalence of overweight and obesity, which is 19.7% (WHO cut-off) and 35% (Asian BMI cut-off) [7–24]. Our findings were in line with another study in hilly urban Nepal, which showed that 34.8% of post-pregnant women (6-12 months) were overweight and obese based on the WHO cut-off, and approximately 57% of women were overweight and obese based on the Asian BMI cut-off [25]. Previous studies in urban Nepal have described the diet of Nepalese women as monotonous, mainly composed of rice with inadequate dietary diversity [26]. The inadequate dietary diversity, the greater availability of food items in an urban area, and increasing per capita energy consumption in the plain (Terai) belt could explain the higher prevalence of overweight and obesity in the study population [27]. Likewise, the more accessible transportation system in an urban area and the consequent decrease in movement and energy expenditure could have contributed to the increased body weight among urban women. Our finding on overweight and obesity (Asian cut-off) was similar to the prevalence of overweight and obesity among Bangladeshi and Sri Lankan women living in an urban area, which was 57.1% and 65%, respectively [28,29]. In comparison to India, the prevalence of overweight and obesity was much higher in our study participants based on both the Asian BMI cut-off (46.3%) and the WHO BMI cut-off (31.3%) [30]. These comparisons indicate that overweight and obesity in our study population is equal to or more than in other South Asian countries, which is a serious concern. Therefore, the concerned authorities in Nepal need to address this issue through appropriate policies and programs.

In the age group studied, we identified an association between increasing age and a higher proportion of overweight and obesity (based on both WHO and Asian BMI cut-offs) among women. It is commonly observed that women of late reproductive age are heavier than younger women. Our finding is supported by the national data, which has shown that Nepalese women in the age group of 35 to 49 years and 25 to 35 years were twice more likely to be overweight and obese than the younger age (15 to < 25 years) group [24]. The significant interaction between age and physical activity level in the unadjusted model showed that physical activity level in the age group 40-49 years could reduce the risk for overweight and obesity. No research is available on the interaction between age and physical activity in overweight and obesity in adults. Therefore, future research with a large sample size needs to be done to understand the association of physical activity with overweight and obesity based on the age group. Interestingly, the study discovered that women belonging to advantaged ethnic/caste groups were two times more likely to be overweight and obese (based on Asian BMI cut-off) than women belonging to disadvantaged ethnic/caste groups. Due to the differences in categorizing ethnic groups/castes between studies, it is difficult to compare our findings with those of other studies. Sutradhar et al. (2021) indicated that people belonging to the disadvantaged categories, such as the Muslims and Terai middle caste, were less likely to be overweight and obese than those in the advantaged categories (*Brahmin/Chhetri*) [8]. There are no studies on dietary intake, ethnicity, and overweight and obesity among Nepalese women. However, it could be speculated that women in the advantaged ethnic group might had greater access to a higher-energy diet than disadvantaged women. This speculation could be supported by the study, which indicated that inadequate food consumption was higher in the disadvantaged group compared to the advantaged group [31].

In Nepal, cultural practices often burden women with dual responsibilities of managing household work and working outside the home, such as office jobs and self-run businesses [32]. Besides, many women are still engaged in some level of agricultural work outside their usual household duties, even in an urban area. These reasons could justify the low prevalence of physical inactivity level (<5000 steps/day) in this study population. The prevalence of physical inactivity (< 600 MET-mins/day) assessed by GPAQ was much higher in other studies conducted in sub-urban areas of Nepal (43.3%) and in urban India (52.1%) [15–33]. These differences in physical inactivity could be attributed to the different study populations and the use of subjective physical activity measurement tools. The limitation of subjective physical activity measurement tools in accurately capturing the physical activity data is well-known in the literature [34]. This can also be illustrated by the weak correlation (r = 0.35) between step counts/day and MET-mins/day observed in our data. Other studies conducted in more than nine countries and a study in Vietnam showed a similar finding of poor to a fair correlation between GPAQ and pedometer/accelerometer assessed physical activity [35–37]. The weak correlation might have been influenced by factors such as limited understanding of terms utilized in GPAQ, like ‘moderate’ and ‘vigorous’ activity, the definition of continuous 10-minute intervals, the possibility of detecting low-intensity steps by the pedometer, and the potential inability of pedometer to detect steps related to labor-intensive activities involving standing with minimal walking. The most important difference between the two assessment methods is the time integration: step counts (in this case) assessed the actual physical activity in the last three consecutive days, and GPAQ assessed physical activity during a fictional ‘typical week.’ The different time integration suggests that a perfectly positive correlation could not be achieved, but with such a low correlation, it is questionable if GPAQ studies are of any use at all when we now have access to objective physical activity measurement tools.

According to WHO, the NCD-related deaths in Nepal have increased from around 87,700 in 2000 to 121,100 in 2016 [38]. Around 49% of premature deaths in Nepal can be attributed to NCDs and NCD-related conditions [39]. Considering the greater prevalence of overweight, obesity, and physical inactivity in sub-urban Nepal, it is high time to implement appropriate preventative policies to reduce these NCDs’ risk factors. We also suggest that research studies be developed to collect data on women’s dietary behavior, such as dietary diversity, total energy consumption, total red meat consumption, and access to junk foods. Dietary studies will help to understand the correlates of the high prevalence of overweight and obesity and whether dietary intervention is equally important to implement along with physical activity interventions.

We believe our data have strong implications for understanding the physical activity and overweight/obesity levels of suburban women in Eastern Nepal. Authorities need to identify and take appropriate actions locally – regarding both diet and physical activity – to curb physical inactivity and overweight and obesity in suburban women in Eastern Nepal.

### Strengths and limitations

The main strength of the study is that it was a community-based study using an objective physical activity measurement tool (pedometer) to measure physical inactivity among women. The usefulness of objective assessments, including a pedometer, outweighs that of questionnaires, such as GPAQ, with less subjective bias [40]. Several concepts related to GPAQ are difficult to comprehend for respondents in Nepal, for instance, ‘10-minute bouts’, ‘heavy breathing,’ and ‘moderate physical activity.’ This may bias the physical activity assessment towards either overestimation or underestimation in women.

One of the drawbacks of this study was that the pedometer might not have captured non-ambulatory activities. Additionally, the pedometer was not designed to track wearing time, and step counting was limited to three days for feasibility reasons. However, the researcher cross-checked participants and their family members to confirm whether participants wore the pedometer the whole time. If participants did not wear them for the three valid days, they were asked if they would wear them again. Participants were deemed non-response, if they would not agree to wear the pedometers again.

Furthermore, only women of the reproductive age group in the plain area of Eastern Nepal were studied, which restricts the generalizability of study findings to women from the hilly/mountainous region of Eastern Nepal. However, the study aimed to study reproductive-aged women in plain Eastern Nepal, and the use of the random sampling technique allows for extrapolation of the result to reproductive-aged women of that study area, i.e., the plain region of Eastern Nepal. We did not measure the dietary habits of women, which limited our ability to control for the confounding effect of diet on overweight and obesity. Lastly, using standardized anthropometrics tools to measure body weight and height ensured the valid measurement of BMI and the valid representation of overweight and obesity among women in Eastern Nepal.

## Conclusions

Our cross-sectional study revealed a high prevalence of overweight and obesity among women of Eastern Nepal, which needs to be addressed. The wide range of physical activity levels suggests a need for more studies on physical activity in a larger group (wider age group and both sexes) and possibly with longer assessment periods. Objective physical activity measurement tools need to be prioritized over GPAQ to quantify physical activity. Increasing age up to 40-49 years, belonging to an advantaged caste, being married, and being a housewife were all positively associated with being overweight and obese. Furthermore, housewives/unemployed women and women from higher socioeconomic status were more likely to be physically inactive. These findings highlight the importance of developing targeted intervention strategies, particularly focusing on older women, unemployed and housewives, and women from higher socioeconomic backgrounds, to effectively address and combat adult overweight obesity and physical inactivity.

## Supporting information

S1 Table Distribution of WHO body mass index (BMI) categories by risk factors, N=330. (DOCX)

S2 Table Distribution of Asian body mass index (BMI) categories by risk factors, N=330. (DOCX)

S3 Table Distribution of physical activity levels (based on step counts) by risk factors, N=330. (DOCX)

S4 Table Distribution of physical inactivity levels (based on MET-minutes/week) by risk factors, N=330. (DOCX)

S1 Checklist PLOS’ questionnaire on inclusivity in global research. (DOCX)

S1 Data Dataset in.sav format. (SAV)
