# Supplementary material for: Overweight, obesity and physical inactivity among women of reproductive age in Eastern Nepal: a cross-sectional community-based study
Source: PLOS Glob Public Health. 2025 Mar 19;5(3):e0004360. doi: 10.1371/journal.pgph.0004360 (PMC11922225; doi:10.1371/journal.pgph.0004360)
Supplement: S1 Table — (DOCX) [file pgph.0004360.s001.docx]

S1 Table: Distribution of WHO body mass index categories by risk factors, N=330

| Characteristics | Underweight  (BMI < 18.5 kg/m^2^)  n (%) | Normal  (BMI ≥ 18.5 < 25 kg/m^2^)  n (%) | Overweight  (BMI ≥ 25 < 30 kg/m^2^)  n (%) | Obesity  BMI ≥ 30 kg/m^2^  n (%) | Total |
| --- | --- | --- | --- | --- | --- |
| Total | 32 (9.7) | 159 (48.2) | 116 (35.2) | 22 (6.7) | 330 |
| Age (years)  18-29  30-39  40-49 | 16 (13.6)  8 (7.3)  9 (8.7) | 73 (61.9)  41 (37.6)  45 (43.7) | 24 (20.3)  53 (48.6)  37 (35.9) | 5 (4.2)  7 (6.4)  12 (11.7) | 118  109  103 |
| Ethnic/ Caste groups  Disadvantaged^1^  Advantaged^2^ | 26 (15.9)  7 (4.2) | 80 (49.1)  79 (47.3) | 49 (30.7)  65 (38.9) | 8 (4.9)  16 (9.6) | 163  167 |
| Marital status  Others  Married | 7 (15.6)  26 (9.1) | 27 (60.0)  132 (46.3) | 10 (22.2)  104 (36.5) | 1 (2.2)  23 (8.1) | 45  285 |
| Occupational status  Manual (labor/ agriculture)  Unemployed/ housewives  Non-manual^3^ | 10 (19.2)  14 (8.8)  9 (7.6) | 30 (57.7)  74 (46.3)  55 (46.6) | 10 (19.2)  58 (36.3)  46 (38.9) | 2 (3.8)  14 (8.8)  8 (6.8) | 52  160  118 |
| Schooling years  Up to nine years  Ten years and above | 20 (14.1)  13 (6.9) | 64 (45.1)  95 (50.5) | 49 (34.5)  65 (35.6) | 9 (6.3)  15 (7.9) | 142  188 |
| Socio-economic tertiles  Lowest  Middle  Top | 18 (16.4)  10 (9.1)  5 (4.5) | 56 (50.9)  49 (44.5)  54 (49.1) | 33 (30.0)  43 (39.1)  38 (34.5) | 3 (2.7)  8 (7.3)  13 (11.8) | 110  110  110 |

^1^ All ethnic groups except upper castes and relatively advantaged *Janajatis;* ^2^ Upper castes and relatively advantaged *Janajatis*

^3^ Includes self-employed, students and office workers
