# Supplementary material for: Overweight, obesity and physical inactivity among women of reproductive age in Eastern Nepal: a cross-sectional community-based study
Source: PLOS Glob Public Health. 2025 Mar 19;5(3):e0004360. doi: 10.1371/journal.pgph.0004360 (PMC11922225; doi:10.1371/journal.pgph.0004360)
Supplement: S2 Table — (DOCX) [file pgph.0004360.s002.docx]

S2 Table: Distribution of Asian body mass index (BMI) categories by risk factors, N=330

| Characteristics | BMI ≥ 23 kg/m^2^  n (%) | BMI < 23 kg/m^2^  n (%) | Underweight  (BMI < 18.5 kg/m^2^)  n (%) | Normal  (BMI ≥ 18.5 < 23 kg/m^2^)  n (%) | Overweight  (BMI ≥ 23 < 27.5 kg/m^2^)  n (%) | Obesity  BMI ≥ 27.5 kg/m^2^  n (%) | Total |
| --- | --- | --- | --- | --- | --- | --- | --- |
| Total | 195 (59.1) | 135 (40.9) | 33 (10.0) | 98 (29.7) | 132 (40.0) | 67 (20.3) | 330 |
| Age (years)  18-29  30-39  40-49 | 48 (40.7)  75 (68.8)  72 (69.9) | 70 (59.3)  34 (31.2)  31 (30.1) | 16 (13.6)  8 (7.3)  9 (8.7) | 54 (45.8)  26 (23.9)  22 (21.4) | 35 (29.7)  43 (39.4)  50 (48.5) | 13 (11.0)  32 (29.4)  22 (21.4) | 118  109  103 |
| Ethnic/ caste groups  Disadvantaged^1^  Advantaged^2^ | 76 (46.63)  119 (71.3) | 48 (29.4)  87 (52.1) | 26 (16.0)  7 (4.2) | 61 (37.4)  41 (24.6) | 47 (28.8)  81 (48.5) | 29 (17.8)  38 (22.8) | 163  167 |
| Marital status  Others  Married | 17 (37.8)  178 (62.4) | 28 (62.2)  107 (37.5) | 7 (15.6)  26 (9.1) | 21 (46.7)  81 (28.4) | 13 (28.9)  115 (40.4) | 4 (8.9)  63 (22.1) | 45  285 |
| Occupational status  Manual (labor/ agriculture)  Unemployed/ housewives  Non-manual^3^ | 21 (40.4)  103 (64.3)  71 (60.2) | 31 (59.6)  57 (35.6)  47 (39.8) | 10 (19.2)  14 (8.8)  9 (7.6) | 21 (40.4)  43 (26.9)  38 (32.2) | 14 (26.9)  68 (42.5)  46 (39.0) | 7 (13.5)  35 (21.9)  25 (21.2) | 52  160  118 |
| Schooling years  Up to nine years  Ten years and above | 113 (79.5)  82 (43.6) | 75 (52.8)  60 (31.9) | 20 (14.1)  13 (6.9) | 37 (26.1)  61 (32.4) | 58 (40.8)  74 (39.4) | 27 (19.0)  40 (21.3) | 142  188 |
| Socio-economic tertiles  Lowest  Middle  Top | 49 (25.1)  67 (34.4)  79 (40.5) | 61 (45.2)  43 (31.9)  31 (23.0) | 18(16.4)  10 (9.1)  5 (4.5) | 43 (39.1)  33 (30.0)  26 (23.6) | 33 (30.0)  43 (39.1)  52 (47.3) | 16 (14.5)  24 (21.8)  27 (24.5) | 110  110  110 |

^1^ All ethnic groups except upper castes and relatively advantaged *Janajatis;* ^2^ Upper castes and relatively advantaged *Janajatis*

^3^ Includes self-employed, students and office workers
