# Supplementary material for: Overweight, obesity and physical inactivity among women of reproductive age in Eastern Nepal: a cross-sectional community-based study
Source: PLOS Glob Public Health. 2025 Mar 19;5(3):e0004360. doi: 10.1371/journal.pgph.0004360 (PMC11922225; doi:10.1371/journal.pgph.0004360)
Supplement: S3 Table — (DOCX) [file pgph.0004360.s003.docx]

S3 Table: Distribution of physical activity levels (based on step counts) by risk factors, N=330

| Characteristics | Physically inactive  (< 5000 steps/day)  n (%) | Low physical activity  (5000-7499 steps/day)  n (%) | Somewhat active  (7500-9999 steps/day)  n (%) | Active  (10000-11999 steps/day) | Highly active  (> 12000 steps/day) | Total |
| --- | --- | --- | --- | --- | --- | --- |
| Total | 46 (13.9) | 85 (25.6) | 104 (31.3) | 38 (11.4) | 57 (17.2) | 330 |
| Age (years)  18-29  30-39  40-49 | 18 (15.3)  17 (15.6)  11 (10.7) | 33 (28.0)  29 (26.6)  23 (22.3) | 44 (37.3)  31 (28.4)  29 (28.2) | 14 (11.9)  10 (9.2)  14 (13.6) | 9 (7.6)  22 (20.2)  26 (25.2) | 118  109  103 |
| Ethnic/ Caste groups  Disadvantaged^1^  Advantaged^2^ | 19 (11.7)  27 (16.2) | 44 (27.0)  41 (24.6) | 49 (30.1)  55 (32.9) | 20 (12.3)  18 (10.8) | 31 (19.0)  26 (15.6) | 163  167 |
| Marital status  Others  Married | 10 (22.2)  36 (12.6) | 13 (28.9)  72 (25.3) | 13 (28.9)  91 (31.9) | 5 (11.1)  33 (11.6) | 4 (8.9)  53 (18.6) | 45  285 |
| Occupational status  Manual (labor/ agriculture)  Unemployed/ housewives  Non-manual^3^ | 2 (3.8)  28 (17.5)  16 (13.6) | 8 (15.4)  42 (26.3)  35 (29.7) | 13 (25.0)  48 (30.0)  43 (36.4) | 6 (11.5)  20 (12.5)  12 (10.2) | 23 (44.2)  22 (13.8)  12 (10.2) | 52  160  118 |
| Schooling years  Up to nine years  Ten years and above | 15 (10.6)  31 (16.5) | 33 (23.2)  52 (27.7) | 39 (27.5)  65 (34.6) | 17 (12.0)  21 (11.2) | 38 (26.8)  19 (10.1) | 142  188 |
| Socio-economic tertiles  Lowest  Middle  Top | 13 (11.8)  9 (8.2)  24 (21.8) | 26 (23.6)  30 (27.3  29 (26.4) | 25 (22.7)  44 (40.0)  35 (31.8) | 20 (18.2)  7 (6.4)  11 (10.0) | 26 (23.6)  20 (18.2)  11 (10.0) | 110  110  110 |

^1^ All ethnic groups except upper castes and relatively advantaged *Janajatis;* ^2^ Upper castes and relatively advantaged *Janajatis*

^3^ Includes self-employed, students and office workers
