# Supplementary material for: Overweight, obesity and physical inactivity among women of reproductive age in Eastern Nepal: a cross-sectional community-based study
Source: PLOS Glob Public Health. 2025 Mar 19;5(3):e0004360. doi: 10.1371/journal.pgph.0004360 (PMC11922225; doi:10.1371/journal.pgph.0004360)
Supplement: S4 Table — (DOCX) [file pgph.0004360.s004.docx]

S4 Table: Distribution of physical inactivity levels (based on MET-minutes/week) by risk factors, N=330

| Characteristics | Low physical activity  (< 600 METmins/day)  n (%) | Moderate physical activity  (600-2999 METmins/day)  n (%) | High physical activity  (≥ 3000 METmins/day)  n (%) | Total |
| --- | --- | --- | --- | --- |
| Total | 76 (23) | 180 (54.5) | 74 (22.4) | 330 |
| Age (years)  18-29  30-39  40-49 | 37 (31.4)  21 (19.3)  18 (17.5) | 65 (55.1)  62 (56.9)  53 (51.5) | 16 (13.6)  26 (23.9)  32 (31.1) | 118  109  103 |
| Ethnic/ Caste groups  Disadvantaged^1^  Advantaged^2^ | 26 (16.0)  50 (29.9) | 94 (57.7)  86 (51.5) | 43 (26.4)  31 (18.6) | 163  167 |
| Marital status  Others  Married | 21 (46.7)  55 (19.3) | 20 (44.4)  160 (56.1) | 4 (8.9)  70 (24.6) | 45  285 |
| Occupational status  Manual (labor/ agriculture)  Unemployed/ housewives  Non-manual^3^ | 1 (1.9)  39 (24.4)  36 (30.5) | 13 (25.0)  98 (61.3)  69 (58.5) | 38 (73.1)  23 (14.4)  13 (11.0) | 52  160  118 |
| Schooling years  Up to nine years  Ten years and above | 18 (12.7)  58 (30.9) | 75 (52.8)  105 (55.9) | 49 (34.5)  25 (13.3) | 142  188 |
| Socio-economic tertiles  Lowest  Middle  Top | 10 (9.1)  27 (24.5)  39 (35.4) | 61 (55.5)  62 (56.4)  57 (51.8) | 39 (35.5)  21 (19.1)  14 (12.7) | 110  110  110 |

^1^ All ethnic groups except upper castes and relatively advantaged *Janajatis;* ^2^ Upper castes and relatively advantaged *Janajatis*

^3^ Includes self-employed, students and office workers
